# The prognostic significance of pretreatment squamous cell carcinoma antigen levels in cervical cancer patients treated by concurrent chemoradiation therapy and a comparison of dosimetric outcomes and clinical toxicities between tomotherapy and volumetric modulated arc therapy

**DOI:** 10.1186/s13014-022-02063-w

**Published:** 2022-05-12

**Authors:** Yuan-Kai Cheng, Shih-Hsun Kuo, Heng-Hsuan Yen, Jing-Hu Wu, Yu-Chieh Chen, Ming-Yii Huang

**Affiliations:** 1 Department of Radiation Oncology, Kaohsiung Medical University Hospital, Kaohsiung, Taiwan; 2 Cancer Center, Kaohsiung Medical University Hospital, Kaohsiung, Taiwan; 3 Department of Radiation Oncology, Faculty of Medicine, College of Medicine, Kaohsiung Medical University, Kaohsiung, Taiwan; 4 Department of Obstetrics and Gynecology, Kaohsiung Medical University Hospital, Kaohsiung Medical University, Kaohsiung, Taiwan; 5 Department of Radiation Oncology, Kaohsiung Medical University Hospital, Kaohsiung Medical University, No. 100 Tzyou 1st Road, Kaohsiung, 80708 Taiwan

**Keywords:** Cervical cancer, SCC Ag, Volumetric modulated arc therapy, Tomotherapy, Diarrhea, Rectum

## Abstract

**Background:**

To analyze the prognostic factors associated with stage IB-IVA cervical cancer in patients who underwent concurrent chemoradiation therapy (CCRT) and to compare the clinical toxicities and dosimetric parameters of organs at risk between the different radiotherapy techniques.

**Methods:**

This retrospective study enrolled 93 patients with stage IB-IVA cervical cancer who underwent definitive CCRT between April 2009 and December 2017. Nine patients (9.7%) received 3DCRT, 43 patients (46.2%) underwent VMAT, and 41 patients (44.1%) received tomotherapy, and all of them followed by brachytherapy using a 2D planning technique. The treatment outcomes and related prognostic factors were analyzed. We also compared the clinical toxicities and dosimetric parameters between the different techniques used for the last 30 patients.

**Results:**

With a median follow-up of 52.0 months, the 5-year overall survival (OS), progression-free survival (PFS), locoregional recurrence–free survival (LRRFS), and distant metastases–free survival (DMFS) were analyzed. In a Cox proportional hazards regression model, pretreatment SCC Ag > 10 ng/mL was a significant prognostic factor for PFS (hazard ratio [HR] 2.20; 95% confidence interval [CI] 1.03–4.70; *P* = 0.041), LRRFS (HR, 3.48; 95% CI 1.07–11.26; *P* = 0.038), and DMFS (HR 2.80; 95% CI 1.02–7.67; *P* = 0.045). Increasing the rectal volume receiving a radiation dose exceeding 30 Gy (V_30_ of rectum; odds ratio [OR] 1.15; 95% CI 1.10–1.30; *P* = 0.03) was associated with a higher possibility of ≥ Grade 2 acute radiation therapy (RT)-related diarrhea. The median rectal V_30_ values were 56.4%, 97.5%, and 86.5% for tomotherapy, 3-dimensional conformal radiation therapy (3DCRT), and volumetric modulated arc therapy (VMAT), respectively (*P* < 0.001). In addition, the chance of experiencing ≥ Grade 2 acute diarrhea were 10.0%, 66.7%, and 54.5% for tomotherapy, 3DCRT, and VMAT, respectively (*P* = 0.029).

**Conclusions:**

Patients with pretreatment SCC Ag ≤ 10 ng/mL have better PFS, LRRFS, and DMFS than those with pretreatment SCC Ag > 10 ng/mL. The rectal V_30_ is a significant predictor of severe acute diarrhea. Tomotherapy significantly decreased the rectal V_30_, reducing the severity of acute RT-related diarrhea during external beam RT.

*Trial registration* This study was approved by the institutional review board at Kaohsiung Medical University Hospital. The registration number is KMUHIRB-E(I)-20190054 and retrospectively registered on 2019/3.

## Background

Cervical cancer is the fourth most common cancer type among women [1]. Despite the development of prophylactic vaccines, cervical cancer remains a major cause of mortality worldwide, particularly in low socioeconomic regions [2]. Concurrent chemoradiation therapy (CCRT) for non-surgical patients with cervical cancer plays an important role in radical therapy. External beam radiation therapy (EBRT) administered using 3-dimensional conformal radiation therapy (3DCRT) is a commonly used cervical cancer treatment method. However, radiation therapy (RT)-related acute and late toxicities are well-known issues, including the development of colitis, diarrhea, cystitis, frequent urination, dysuria, and proctitis.

Increasingly, intensity-modulated radiotherapy (IMRT), volumetric modulated arc therapy (VMAT), and tomotherapy have become more commonly used RT methods over the past few decades. Comparisons of clinical results between 3DCRT, VMAT, and tomotherapy among patients with head and neck cancer have been well described [3, 4]. Considerable studies have also examined the dosimetric differences among 3DCRT, VMAT, and tomotherapy in patients with cervical cancer. Some single-center and multi-center series examining postoperative RT have described favorable toxicity profiles associated with the use of IMRT [5, 6]. However, disparities in the clinical results among these techniques are rarely reported. Thus, we compared the clinical outcomes across various techniques applied to patients with non‐distant metastatic cervical cancer who underwent definitive CCRT and examined prognostic factors.

## Methods

### Patients

We enrolled patients diagnosed with cervical cancer, classified as stages IB to IVA according to the International Federation of Gynecology and Obstetrics (FIGO) staging system, between April 2009 and December 2017. None of the enrolled patients had distant metastases at treatment onset, and all patients received radical CCRT. The exclusion criteria for this study included any history of prior malignancy before treatment, any history of prior radiotherapy, and Eastern Cooperative Oncology Group (ECOG) performance status > 2.

All patients underwent pretreatment workup and cancer staging using modern approaches, including a physical examination by a gynecologic oncologist, a tumor biopsy, a history review, chest X-ray, abdominal and pelvic computed tomography (CT,) or pelvic magnetic resonance imaging (MRI). Cystoscopy or sigmoidoscopy was performed by a specialist to exclude adjacent organ invasion for patients with locally advanced disease. In addition, routine laboratory biomarker studies, including squamous cell carcinoma antigen (SCC Ag), carcinoembryonic antigen (CEA), and cancer antigen 125 (CA125), were measured among the cohort. The median follow-up was 52 months (range 6–137 months). The cancer stage was classified according to the seventh edition of the American Joint Committee on Cancer (AJCC) TNM classification and the 2008 International FIGO staging system for cervical cancer. The retrospective study (KMUHIRB-E(I)-20190054) was approved by the Institutional Review Board (IRB) of Kaohsiung Medical University Hospital, and the need for informed consent was waived by the IRB due to the nature of this study as a chart review.

### Radiotherapy

All patients received a consultation with a radiation oncologist and underwent an evaluation of clinical status to ensure the necessity and safety of radiotherapy. Following bladder preparation, patients were placed in a supine position with cast or cushion immobilization and underwent CT simulation, using a 3–5 mm slice thickness, from the upper edge of the lumbar spine to 5 cm below the lower border of the obturator foramen. For 3DCRT, a four-field box technique was planned using corner shielding in anteroposterior/posteroanterior (AP/PA) portals. The radiation portal fields were designed as follows: (1) superior border: L4–5 interspace, which covers the common iliac lymph nodes; (2) inferior border: 3 cm below the most inferior vaginal involvement, which is often below the inferior obturator foramen and can be as low as the introitus in cases of vaginal tumor extension; and (3) lateral border: 1.5–2 cm outside of the pelvic rim. For the lateral fields, the superior and inferior borders were consistent with the design of the AP/PA portals. The anterior border covered the front of the pubic symphysis, and the posterior edge included the entire sacrum. Pelvic radiotherapy was delivered at 1.8–2.0 Gy per fraction, 1–5 days each week, for a total of 25 fractions comprising 45–50 Gy, followed by the delivery of 5.4–9 Gy in 3–5 fractions delivered by AP/PA portals using a midline block. The superior border of the midline block was the midsacroiliac joint, and the width was 4 cm. If a parametrial tumor persists after 50–54 Gy, the side wall or parametrium may receive up to 60 Gy. For VMAT or tomotherapy, the clinical target volume (CTV) was defined as the gross tumor plus microscopic disease, including the cervix, uterus, upper third of the vagina (or upper half of the vagina, if a gross tumor is involved), the parametrium, and the pelvic nodal drainage. The patients were treated with simultaneous integrated boost doses of 48.6–50.4 Gy, delivered in 1.8 to 2-Gy fractions, to the primary tumor, 54–60 Gy delivered to the pelvic nodal drainage, including parametrial, obturator, internal iliac, external iliac, common iliac and gross lymph nodes, and 45–48 Gy, delivered in 1.6 to 1.8-Gy fractions, to elective nodal regions, such as presacral and paraaortic area. The planning target volume (PTV) was defined as the 8–10 mm margin around the CTV and could be modified according to the clinical condition. Target planning constraints were standardized as follows: (1) the PTV in all directions to receive > 95% of the prescribed dose; (2) volumes receiving more than 110% of the dose prescribed to the PTV were minimized. The typical organs at risk (OARs) included the rectum, bladder, intestine, large bowel, peritoneum, bilateral femoral heads, and the pelvic bone marrow [7]. The external contours of all bones within the pelvis were delineated on the planning CT images, as surrogates for the bone marrow, to enhance the reproducibility and consistency of the contours. The intestine and large bowel contours consisted of the bowel loops from 3 cm superior to the upper border of the PTV to its lowest extent in the pelvis. The dosimetric parameters for OARs were recorded as Vx, which represented the percentage of the organ volume that received X Gy or higher. For the individual patients, the selection of respective EBRT technique was decided by radiation oncologist on the basis of clinical scenario.

### Brachytherapy and concurrent chemotherapy

After EBRT, all patients underwent afterloading brachytherapy, which consisted of high-dose-rate ^192^Ir intracavitary brachytherapy intended to deliver a dose of 4–5 Gy/time to Point A twice per week, with 5 to 6 total treatments. During RT, chemotherapy was concurrently prescribed, consisting of weekly cisplatin for 6 weeks. The regimen was shifted to carboplatin for those patients with impaired renal function and paclitaxel-based treatment for prescribed for patients with locally advanced disease.

### Follow-up and evaluation

In general, the patients returned for a first follow-up visit one month after the completion of treatment, followed by every 2–3 months during the first year and every 3–6 months thereafter. Physical examination including pelvic examination was performed at every follow-up visit. Patients should have follow-up imaging, either abdominal and pelvic CT or pelvic MRI, at least every 3–6 months after the completion of treatment. Chest X-ray is acquired annually at least after treatment. A serum test for tumor markers was performed every 3–6 months after the completion of CCRT. The gynecologic oncologists and radiation oncologists recorded treatment-related toxicity events according to the Common Terminology Criteria of Adverse Events (CTCAE), VERSION 4.03 [28]. Using these criteria, acute complications were defined as those with onset during RT and were assessed once per week during EBRT. Chronic complications were scored retrospectively based on chart records. The overall treatment time (OTT) of RT was defined as the time interval between the first and last date of RT. The primary endpoints were locoregional recurrence–free survival (LRRFS), progression-free survival (PFS), distant metastasis–free survival (DMFS), and overall survival (OS). The length of follow-up was defined as the time from CCRT to the date of death or the last follow-up. Locoregional failure was defined as any recurrent or persistent disease involving the pelvis. Any disease failure outside of the pelvis was defined as a distant failure. Pathological reports, including those associated with surgical intervention, biopsy, and cytology, in addition to radiology reports from radiology examinations, including chest radiography, CT, MRI, technetium-99 bone scintigraphy, or positron emission tomography (PET), were reviewed to determine disease status.

### Statistical analysis

Data were analyzed using SPSS 22.0 software (IBM Corp., Armonk, NY, USA). Dose-volume histograms (DVHs) of the PTVs and the OARs were analyzed accordingly. For PTV, the goal is to encompass the PTV in all directions with the 95% isodose line. To reduce toxicity and optimize OAR doses, DVH constraint was applied to limit maximum dose and dose-volume parameters. OS was defined as the time from primary treatment to the date of death from any cause or the date of the last follow-up. PFS was defined as the time from primary treatment to the date of disease failure at any site or to the date of the last follow-up. LRRFS was defined as the time from primary treatment to the date of locoregional failure or to the date of the last follow-up. DMFS was defined as the time from primary treatment to the date of distal failure or to the date of the last follow-up. LRRFS, PFS, DMFS, OS, and the treatment-related toxicity were analyzed using the Kaplan–Meier method, and the log-rank test was used to calculate differences between groups. Significance was defined as *P* < 0.05.

## Results

### Patients

A total of 93 patients diagnosed with stage IB-IVA cervical cancer were enrolled in this retrospective study. The median age of the retrospective cohort was 61 years (range 34–93 years). Table 1 summarizes the patients’ clinical baseline characteristics, grouped according to the three radiotherapy techniques. Nine patients (9.7%) received 3DCRT, 43 patients (46.2%) underwent VMAT, and 41 patients (44.1%) received tomotherapy. The median EBRT dose was 54 Gy (range 45–64 Gy), and the median equivalent dose in 2-Gy fractions (EQD2) of Point A was 79.8 Gy (range 49.1–93.1 Gy). No significant differences were observed for OTT of RT, clinical T classification, histological type, mean EBRT dose, EQD2 of Point A, pre- and post-treatment SCC Ag, or follow-up duration between the three techniques. One patient in the 3DCRT group and one patient in the VMAT group were lost to follow-up.

**Table 1:** Patient and tumor characteristics for all 93 patients

Characteristics	3DCRT (n = 9)	VMAT (n = 43)	Tomotherapy (n = 41)	*P* value
Age in years, median (range)	48 (34–66)	62 (38–84)	63 (34–89)	0.01
Age (years)				
< 60	8	20	17	0.034
≥ 60	1	23	24	
OTT of RT (days)				
≤ 61	6	21	13	0.091
> 61	3	22	28	
FIGO stage				
I	1	10	3	0.081
II	4	25	22	
III	1	4	11	
IV	3	4	5	
T classification				
T1/T2	6	35	25	0.114
T3/T4	3	8	16	
Nodal classification				
N0	2	32	25	0.011
N1	7	11	16	
Histological type				
Squamous cell carcinoma	8	40	39	0.607
Adenocarcinoma	1	3	1	
Others	0	0	1	
Mean EBRT dose (Gy)	55.1	54.1	54.2	0.516
Mean EQD2 of brachytherapy (Gy)	28.1	29.9	29.8	0.696
EQD2 of Point A (Gy)				
< 81	8	27	32	0.147
≥ 81	1	16	9	
Pretreatment SCC Ag (ng/mL)				
≤ 10	4	23	24	0.722
> 10	5	20	17	
Post-treatment SCC Ag (ng/mL)				
≤ 1.5	8	38	32	0.399
> 1.5	1	5	9	
Median follow-up (months)	43	54	52	0.123

*3DCRT* three-dimensional conformal radiation therapy, *VMAT* volumetric modulated arc therapy, *OTT* overall treatment time, *RT* radiation therapy, *FIGO* the international federation of gynaecology and obstetrics, *EBRT* external beam radiation therapy, *EQD2* equivalent dose in 2-Gy fractions, *SCC Ag* squamous cell carcinoma antigen

### Clinical outcomes and failure patterns

With a median follow-up of 52 months (range 6–137 months), the 5-year OS, PFS, LRRFS, and DMFS were 75.2%, 65.8%, 82.2%, and 74.7% (*P* = 0.07, 0.06, 0.36, and 0.23), respectively. No significant differences in survival outcomes were observed between the three groups. The overall locoregional recurrence rate was 16.1% (15/93), and the majority recurrence pattern was local recurrence (11 patients with local recurrence and 4 patients with regional nodal failure). The distant failure rate was 23.7% (22/93), and the major recurrence sites included the non-regional lymph nodes (7/22), the lung (5/22), and the liver (5/22), with other sites observed less frequently.

**Fig. 1:**
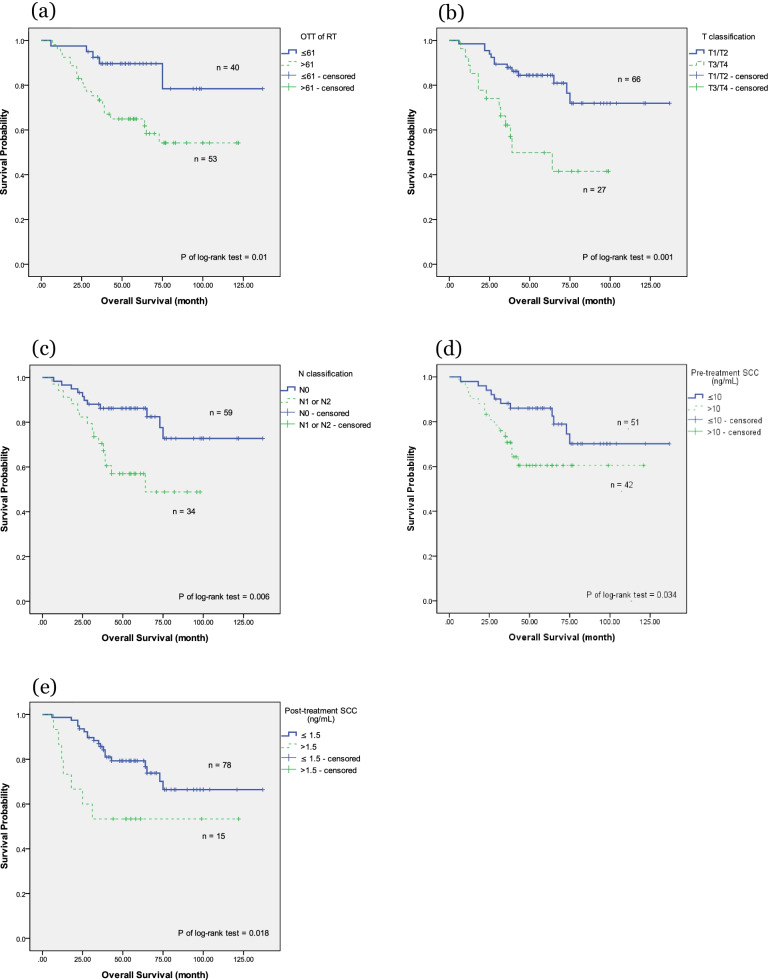
The Kaplan–Meier survival curve of overall survival. Overall survival correlated with **a** overall treatment time of radiation therapy **b** T classification **c** N classification **d** pretreatment serum squamous cell carcinoma antigen (SCC Ag), and **e** post-treatment serum SCC Ag

We further investigated clinical outcomes based on different patient and tumor characteristics, including age (≥ 60 vs. < 60 years), OTT of RT (> 61 days vs. ≤ 61 days), T classification (T1/T2 vs. T3/T4), N classification (nodal negative vs. nodal positive), pretreatment SCC Ag (≤ 10 vs. > 10 ng/mL), post-treatment SCC Ag (≤ 1.5 vs. > 1.5 ng/mL), the EQD2 of Point A (≥ 81 vs. < 81 Gy), and the RT technique (3DCRT vs. VMAT vs. Tomotherapy). In the OS analysis, using the Kaplan–Meier method (Fig. 1), the OTT of RT, T classification, N classification, pretreatment SCC Ag, and post-treatment SCC Ag were significant factors (*P* = 0.01, 0.001, 0.006, 0.034, and 0.018, respectively). Table 2 presents a multivariate analysis of these characteristics. Only an OTT of RT > 61 days and T3/T4 disease were significant factors associated with OS in the Cox proportional hazards regression analysis (hazard ratio [HR] 2.99, 95% confidence interval [CI] 1.03–8.70, *P* = 0.045; and HR 2.97, 95% CI 1.24–7.11, *P* = 0.015, respectively). A post-treatment SCC Ag > 1.5 ng/mL was associated with a lower OS, but did not achieve significance (*P* = 0.069).

**Table 2:** Cox proportional hazards regression analysis for overall survival

Variable	HR	95% CI	*P* value
Age ≥ 60 years	1.91	0.78–4.67	0.156
OTT of RT > 61 days	2.99	1.03–8.70	0.045
T3 or T4 disease	2.97	1.24–7.11	0.015
N1 or N2 disease	2.11	0.87–5.13	0.098
Pretreatment SCC Ag > 10 ng/mL	1.72	0.71–4.17	0.232
Post-treatment SCC Ag > 1.5 ng/mL	2.42	0.93–6.26	0.069
EQD2 of Point A ≥ 81 Gy	0.82	0.34–1.99	0.666
RT technique^a^			0.621
VMAT	0.44	0.08–2.39	0.341
Tomotherapy	0.59	0.12–2.90	0.518

*HR* hazard ratio, *CI* confidence interval, *OTT* overall treatment time, *RT* radiation therapy, *SCC Ag* squamous cell carcinoma antigen, *EQD2* equivalent dose in 2-Gy fractions, *VMAT* volumetric modulated arc therapy

^a^Reference category: 3DCRT, three-dimensional conformal radiation therapy

**Fig. 2:**
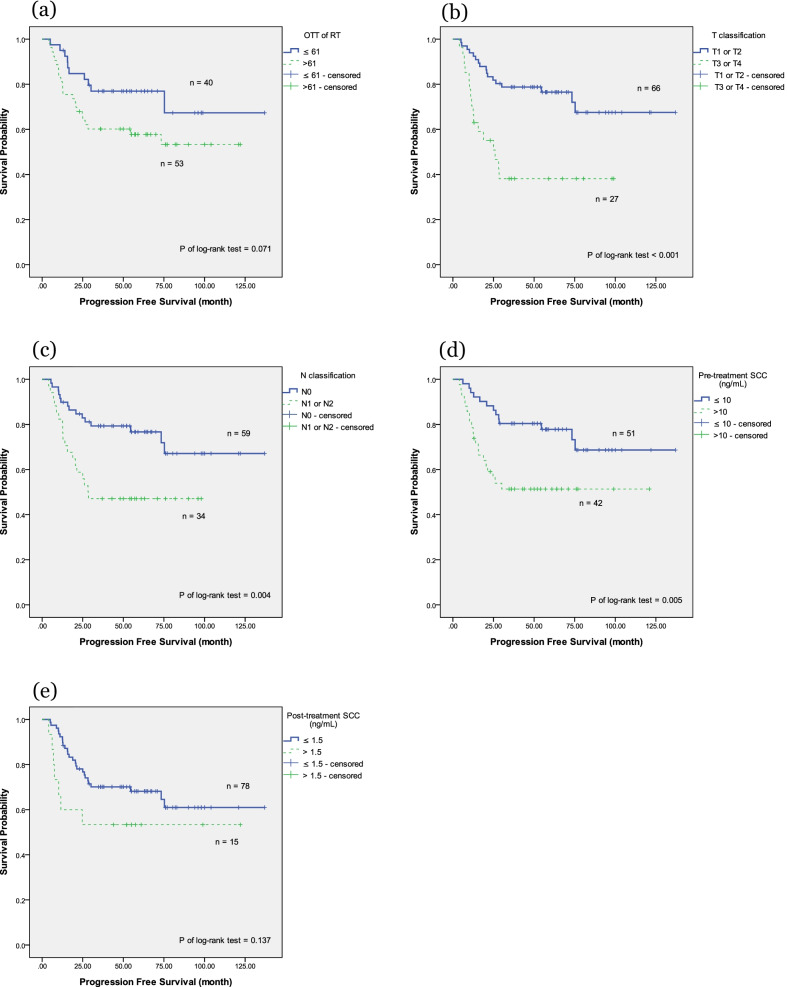
The Kaplan–Meier survival curve of progression-free survival. Progression-free survival correlated with **a** overall treatment time of radiation therapy **b** T classification **c** N classification **d** pretreatment serum squamous cell carcinoma antigen (SCC Ag), and **e** post-treatment serum SCC Ag

In the PFS analysis using the Kaplan–Meier method (Fig. 2), T classification, N classification, and pretreatment SCC Ag were significant factors (*P* ≤ 0.001, 0.004, and 0.005, respectively). The OTT of RT showed an effect on PFS by the log-rank test, but did not achieve significance (*P* = 0.071). Table 3 presents the multivariate analysis of these characteristics. T3/T4 disease, nodal positive, and pretreatment SCC Ag > 10 ng/mL remained significant factors affecting PFS in the Cox proportional hazards regression analysis (HR 2.72, 95% CI 1.30–5.71, *P* = 0.008; HR 2.55, 95% CI 1.15–5.63, *P* = 0.021; and HR 2.20, 95% CI 1.03–4.71, *P* = 0.041, respectively).

**Table 3:** Cox proportional hazards regression analysis for progression-free survival

Variable	HR	95% CI	*P* value
Age ≥ 60 years	1.90	0.84–4.28	0.122
OTT of RT > 61 days	1.61	0.69–3.78	0.273
T3 or T4 disease	2.72	1.30–5.71	0.008
N1 or N2 disease	2.55	1.15–5.63	0.021
Pretreatment SCC Ag > 10 ng/mL	2.20	1.03–4.71	0.041
Post-treatment SCC Ag > 1.5 ng/mL	2.01	0.82–4.96	0.129
EQD2 of Point A ≥ 81 Gy	1.39	0.65–2.97	0.403
RT technique^a^			0.423
VMAT	0.53	0.13–2.08	0.359
Tomotherapy	0.91	0.25–3.33	0.889

*HR* hazard ratio, *CI* confidence interval, *OTT* overall treatment time, *RT* radiation therapy, *SCC Ag* squamous cell carcinoma antigen, *EQD2* equivalent dose in 2-Gy fractions, *VMAT* volumetric modulated arc therapy

^a^Reference category: 3DCRT, three-dimensional conformal radiation therapy

**Fig. 3:**
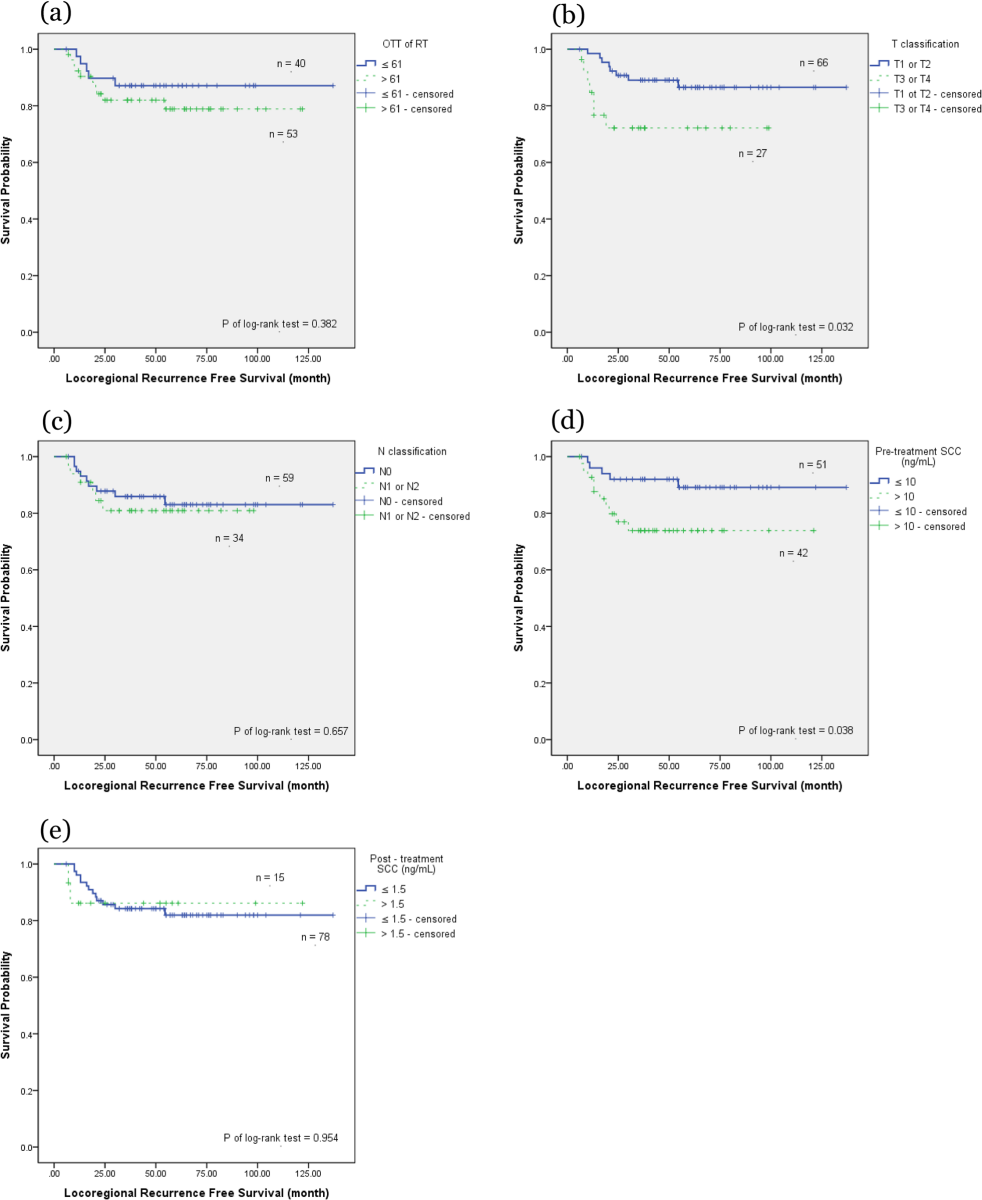
The Kaplan–Meier survival curve of locoregional recurrence-free survival. Locoregional recurrence-free survival correlated with **a** overall treatment time of radiation therapy **b** T classification **c** N classification **d** pretreatment serum squamous cell carcinoma antigen (SCC Ag), **e** and post-treatment serum SCC Ag

In the LRRFS analysis using the Kaplan–Meier method (Fig. 3), only T classification and pretreatment SCC Ag were significant factors (*P* = 0.032 and 0.038, respectively). Table 4 presents the multivariate analysis of these characteristics. Only pretreatment SCC Ag > 10 ng/mL remained significant in the Cox proportional hazards regression analysis (HR 3.48, 95% CI 1.07–11.26, *P* = 0.038). The T3/T4 classification showed an effect on LRRFS but failed to reach significance (*P* = 0.082).

**Table 4:** Cox proportional hazards regression analysis for locoregional recurrence–free survival

Variable	HR	95% CI	*P* value
Age ≥ 60 years	1.18	0.39–3.59	0.775
OTT of RT > 61 days	1.32	0.41–4.29	0.641
T3 or T4 disease	2.64	0.88–7.86	0.082
N1 or N2 disease	0.78	0.24–2.58	0.688
Pretreatment SCC Ag > 10 ng/mL	3.48	1.07–11.26	0.038
Post-treatment SCC Ag > 1.5 ng/mL	0.71	0.15–3.35	0.667
EQD2 of Point A ≥ 81 Gy	1.16	0.35–3.82	0.804
RT technique^a^			0.389
VMAT	0.87	0.09–8.26	0.905
Tomotherapy	2.10	0.24–18.58	0.504

*HR* hazard ratio, *CI* confidence interval, *OTT* overall treatment time, *RT* radiation therapy, *SCC Ag* squamous cell carcinoma antigen, *EQD2* equivalent dose in 2-Gy fractions, *VMAT* volumetric modulated arc therapy

^a^Reference category: 3DCRT, three-dimensional conformal radiation therapy

**Fig. 4:**
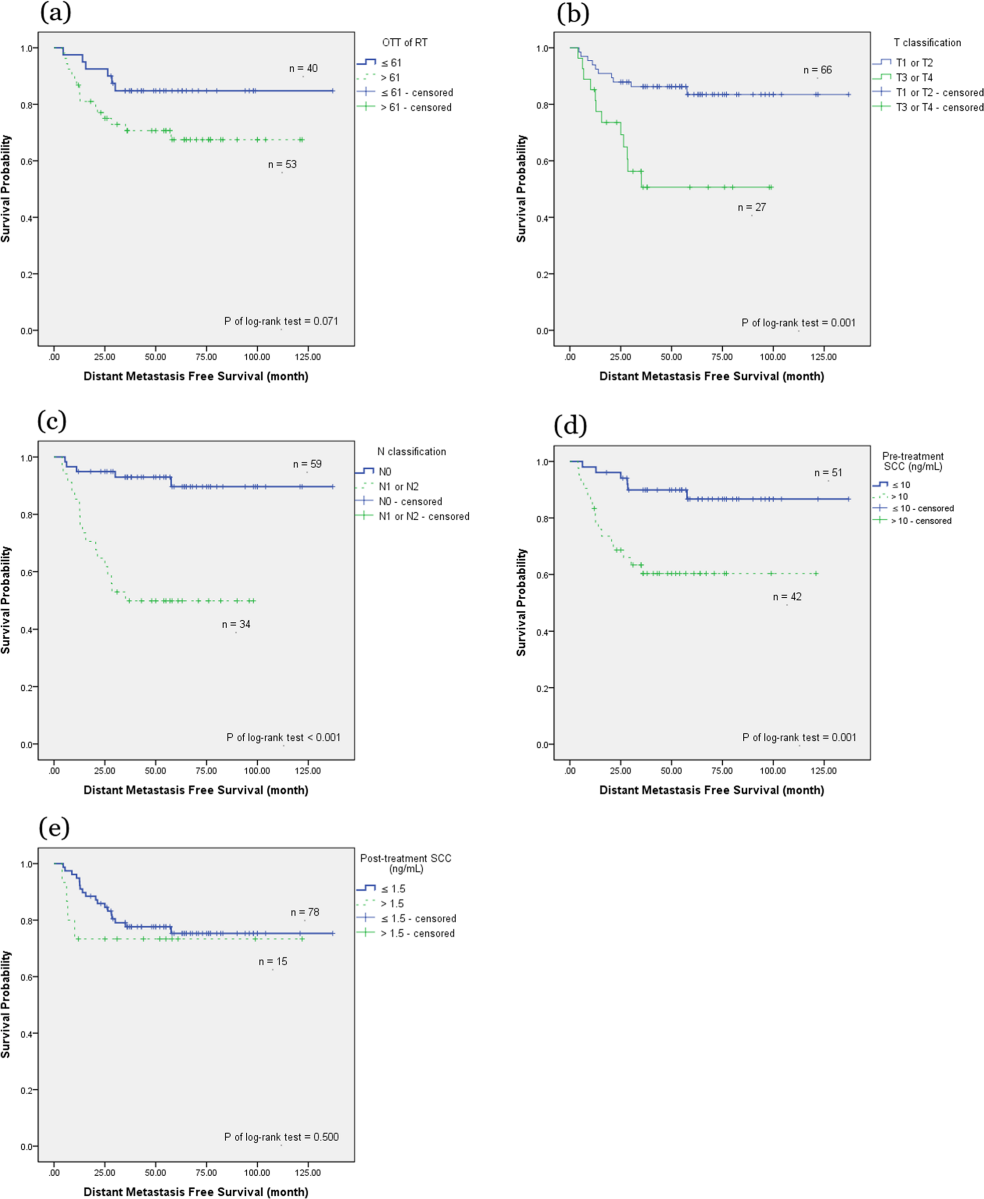
The Kaplan–Meier survival curve of distant metastases-free survival. Distant metastases-free survival correlated with **a** overall treatment time of radiation therapy **b** T classification **c** N classification **d** pretreatment serum squamous cell carcinoma antigen (SCC Ag), and **e** post-treatment serum SCC Ag

In the DMFS analysis using the Kaplan–Meier method (Fig. 4), T classification, N classification, and pretreatment SCC Ag were significant factors (*P* = 0.001, < 0.001, and 0.001, respectively). The OTT of RT showed an effect on DMFS by the log-rank test but failed to reach significance (*P* = 0.071). Table 5 presents the multivariate analysis of these characteristics. All three factors remained significant in the Cox proportional hazards regression analysis (HR 2.88, 95% CI 1.01–8.22, *P* = 0.048; HR 6.17, 95% CI 2.01–18.89, *P* = 0.001; and HR 2.80, 95% CI 1.02–7.67, *P* = 0.045, respectively). OTT of RT > 61 days failed to demonstrate significance following after covariate adjustment (*P* = 0.161).

**Table 5:** Cox proportional hazards regression analysis for distant metastases–free survival

Variable	HR	95% CI	*P* value
Age ≥ 60 years	1.32	0.47–3.65	0.599
OTT of RT > 61 days	2.27	0.72–7.14	0.161
T3 or T4 disease	2.88	1.01–8.22	0.048
N1 or N2 disease	6.17	2.01–18.89	0.001
Pretreatment SCC Ag > 10 ng/mL	2.80	1.02–7.67	0.045
Post-treatment SCC Ag > 1.5 ng/mL	0.94	0.27–3.34	0.926
EQD2 of Point A ≥ 81 Gy	1.32	0.47–3.68	0.601
RT technique^a^			0.662
VMAT	0.67	0.15–2.96	0.596
Tomotherapy	0.51	0.12–2.19	0.368

*HR* hazard ratio, *CI* confidence interval, *OTT* overall treatment time, *RT* radiation therapy, *SCC Ag* squamous cell carcinoma antigen, *EQD2* equivalent dose in 2-Gy fractions, *VMAT* volumetric modulated arc therapy

^a^Reference category: 3DCRT, three-dimensional conformal radiation therapy

**Fig. 5:**
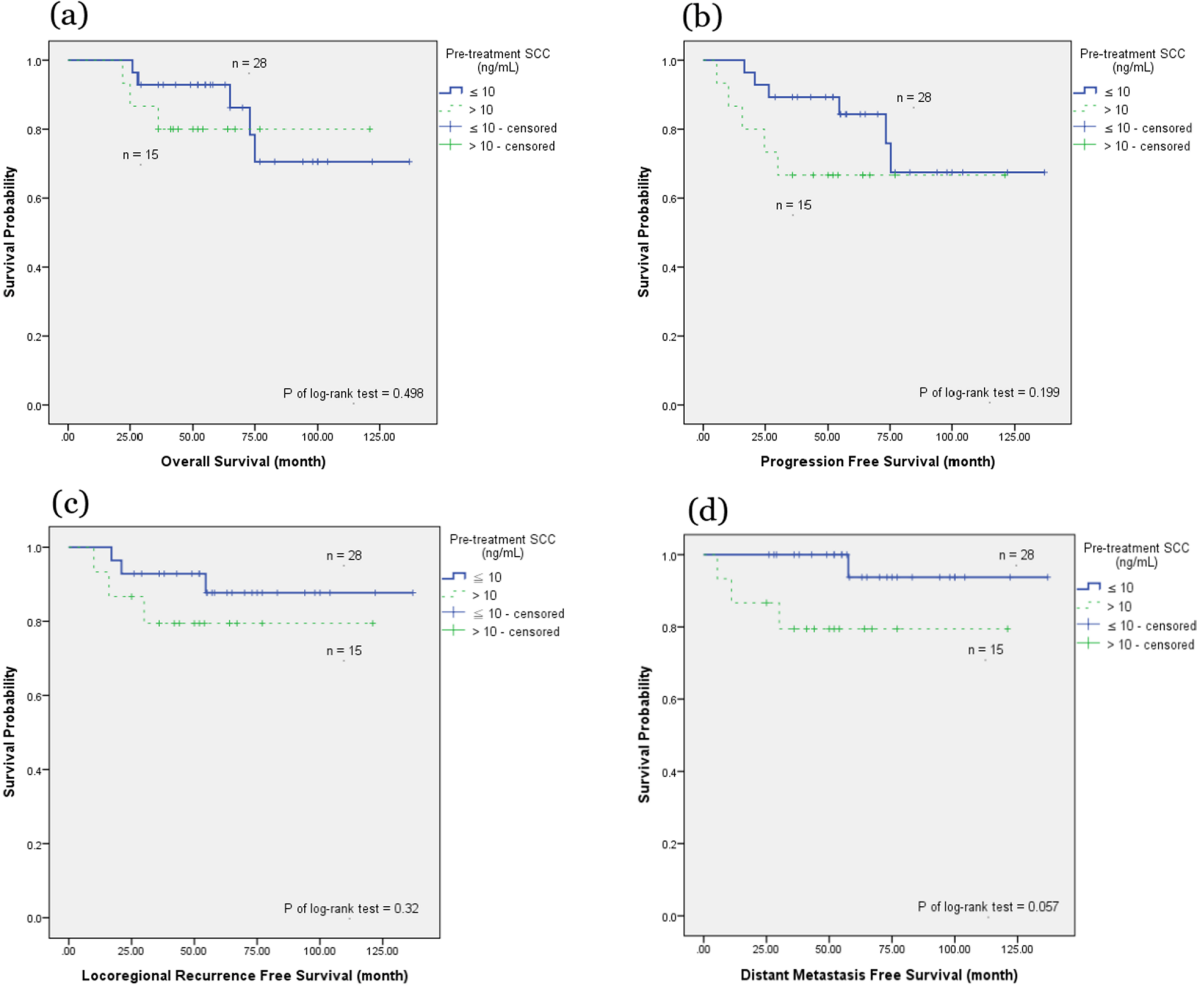
Analysis of the association between survival outcomes and pre-treatment serum SCC in T1/T2N0 subgroup. Pretreatment serum squamous cell carcinoma antigen (SCC Ag) correlated with **a** overall survival, **b** progression-free survival, **c** locoregional recurrence-free survival, **d** and distant metastasis-free survival in the T1/T2N0 subgroup

In the T1/T2N0 subgroup analysis using the Kaplan–Meier method (Fig. 5), pretreatment SCC Ag > 10 ng/mL trended toward worse DMFS but not OS, PFS, or LRRFS. The 5-year DMFS was 93.8% for the group with pretreatment SCC Ag ≤ 10 ng/mL, compared with 79.4% for the group with pretreatment SCC Ag > 10 ng/mL (*P* = 0.057). Furthermore, in the Cox proportional hazards regression analysis (Table 6), pretreatment SCC Ag > 10 ng/mL suggested an increased risk of distant metastasis and nearly reached a significant effect on DMFS (HR 12.4, 95% CI 0.85–181.4, *P* = 0.066). However, all other factors, such as age, OTT of RT, post-treatment SCC Ag, EQD2 of Point A, and RT technique, failed to show significant effects after covariate adjustment (*P* = 0.161, 0.767, 0.863, 0.921, and 0.991, respectively).

**Table 6:** Cox proportional hazards regression analysis for distant metastases–free survival in the T1/T2 N0 subgroup

Variable	HR	95% CI	*P *value
Age ≥ 60 years	5.94	0.49–71.71	0.161
OTT of RT > 61 days	1.48	0.11–19.78	0.767
Pretreatment SCC Ag > 10 ng/mL	12.40	0.85–181.40	0.066
Post-treatment SCC Ag > 1.5 ng/mL	0.001	0.00–1.078E33	0.863
EQD2 of Point A ≥ 81 Gy	1.15	0.077–16.98	0.921
RT technique^a^			0.991
VMAT	227.32	0.00–2.350E178	0.979
Tomotherapy	192.6	0.00–1.995E178	0.980

*HR* hazard ratio, *CI* confidence interval, *OTT* overall treatment time, *RT* radiation therapy, *SCC Ag* squamous cell carcinoma antigen, *EQD2* equivalent dose in 2-Gy fractions, *VMAT* volumetric modulated arc therapy

^a^Reference category: 3DCRT, three-dimensional conformal radiation therapy

### Dosimetric parameters for organs at risk and toxicity

The dosimetric parameters and RT-related toxicity are summarized in Table 7. The relationships between toxicity and OAR doses were analyzed by logistic regression. Due to clinical limitations, only the last 30 patients were able to be analyzed.

**Table 7:** Acute and chronic gastrointestinal toxicity by dosimetric parameters

	CTCAE Grade 2 + acute diarrhea			CTCAE Grade 2 + chronic colitis		
	Odds ratio	95% CI	*P*	Odds ratio	95% CI	*P*
*Colon*						
V_35_	0.88	0.73–1.06	0.182	1.10	0.86–1.41	0.451
V_25_	1.14	0.93–1.40	0.202	0.82	0.60–1.13	0.224
V_15_	0.93	0.83–1.04	0.186	1.05	0.93–1.18	0.448
*Peritoneum*						
V_50.4_	0.80	0.57–1.12	0.198	1.09	0.76–1.56	0.629
V_40_	1.62	0.97–2.71	0.068	0.93	0.63–1.36	0.698
V_30_	0.80	0.54–1.18	0.250	1.25	0.83–1.87	0.287
V_25_	0.99	0.83–1.17	0.892	1.02	0.89–1.16	0.792
*Rectum*						
V_50.4_	1.01	0.92–1.11	0.863	0.97	0.87–1.08	0.576
V_40_	0.92	0.83–1.02	0.116	0.84	0.67–1.06	0.147
V_30_	1.15	1.10–1.30	0.030*	1.14	0.99–1.33	0.073

*CTCAE* common terminology criteria for adverse events, *CI* confidence interval, V_50.4_, V_40_, V_35_, V_30_, V_25_, V_15_ = volume receiving ≥ 50.4, ≥ 40, ≥ 35, ≥ 30, ≥ 25, ≥ 15 Gy, respectively

*Statistically significant

The dose delivered to the colon did not affect the likelihood of experience Grade 2 or worse acute diarrhea. The colon V_35_, V_25_, and V_15_ values were analyzed, and no correlation was observed between these dosimetric parameters and the occurrence of Grade 2 or worse acute diarrhea. We also analyzed the dosimetric parameters for the peritoneum and noted a trend toward the increased occurrence of Grade 2 or worse acute diarrhea with an increasing peritoneum V_40_ value (odds ratio [OR] 1.62, 95% CI 0.97–2.71, *P* = 0.068) but not for the peritoneum V_50.4_, V_30_, or V_25_ values. The dosimetric parameters for the rectum showed a significant increase in the occurrence of Grade 2 or worse acute diarrhea with an increasing rectum V_30_ value (OR 1.15, 95% CI 1.10–1.30, *P* = 0.030) but not for the rectum V_50.4_ and V_40_ values.

The doses delivered to the colon and peritoneum did not affect the likelihood of Grade 2 or worse colitis. The colon V_35_, V_25_, and V_15_ and the peritoneum V_50.4_, V_40_, V_30_, and V_25_ values were analyzed, and no correlations were observed between these dosimetric parameters and the occurrence of Grade 2 or worse colitis. However, we found a trend toward the increased likelihood of Grade 2 or worse colitis correlated with an increasing rectum V_30_ value (OR 1.14, 95% CI 0.99–1.33, *P* = 0.073), although this did not achieve significance. Except for the rectum V_30_ value, the occurrence of Grade 2 or worse colitis was not correlated with any other dosimetric parameters for the rectum.

### Dosimetric parameters of organs at risk and RT technique

**Fig. 6:**
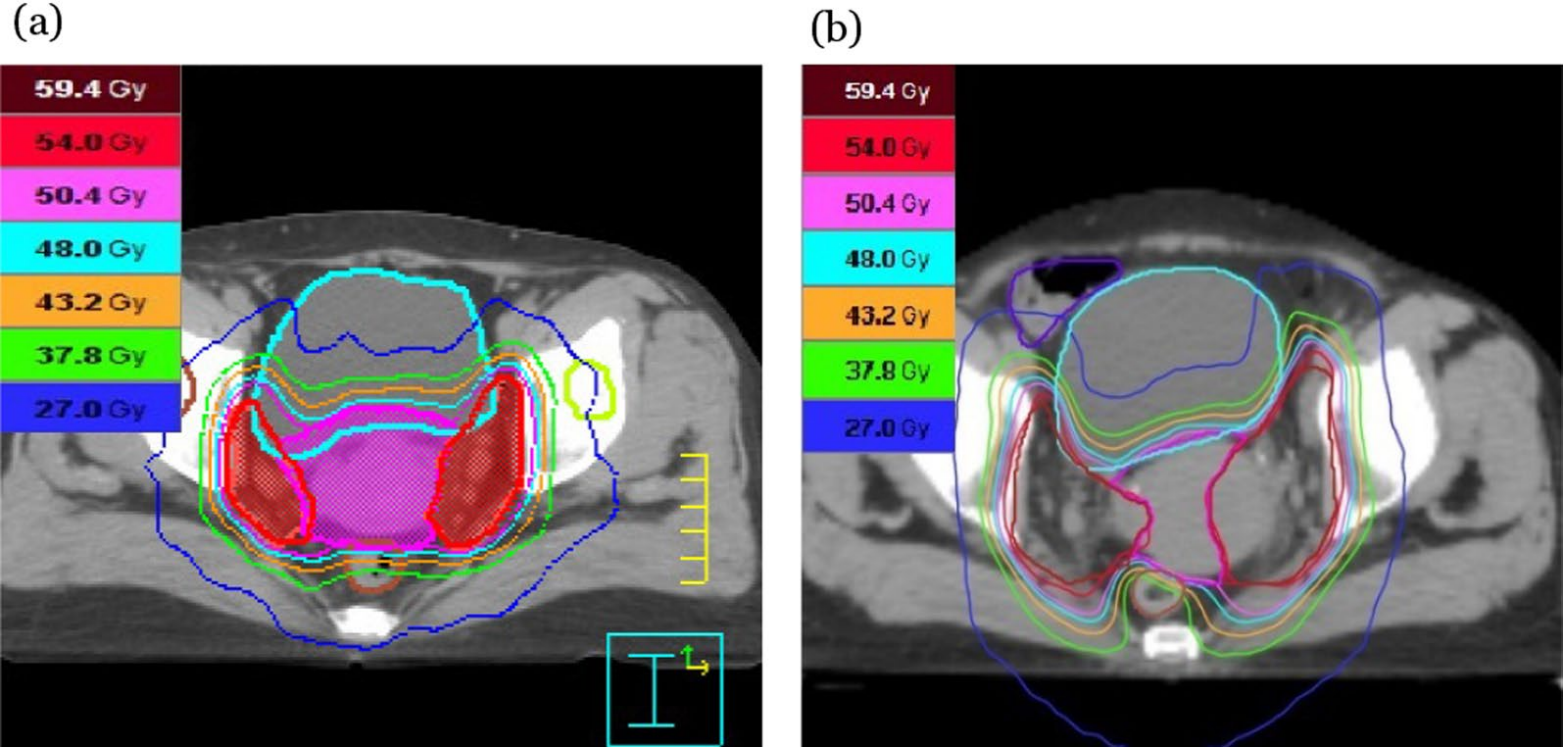
The isodose distributions of patients with cervical cancer treated by VAMT and Tomotherapy. The isodose distributions for **a** a T2N0 patient treated with volumetric modulated arc therapy and **b** a T3N0 patient treated with tomotherapy. The thick brown and light blue lines represented the border of rectum and bladder, respectively. The thin red, pink, light blue, orange, green lines represented the dose curves of 54, 50.4, 48, 43.2 and 37.8 Gy, respectively. Tomotherapy provided significantly less rectal volume exposed to 37.8 Gy

To compare differences in the radiation exposure for OARs between the 3 treatment plans, the last 30 patients were analyzed, including 9 patients (30%) in the 3DCRT group, 11 patients (36.7%) in the VMAT group, and 10 patients (33.3%) in the tomotherapy group. Since we were also interested in OARs and toxicity difference between the 3 treatment plans, our group initiated the comparison of dosimetric outcomes and clinical toxicities in the mid-term of study and led to only the last 30 patients were included. Furthermore, the improved conformality achievable with IMRT can potentially mitigate adverse effects and contribute to the low utilization of 3DCRT. Table 8 presents the dosimetric comparisons for OARs across the 3 treatment plans. Three dosimetric parameters were analyzed for the colon, including the V_35_, V_25_, and V_15_ values. 3DCRT was associated with higher colon V_35_ and V_25_ values than VMAT and tomotherapy (*P* = 0.002 and 0.020, respectively). However, the colon V_15_ values were similar across the three groups. In addition, no dosimetric differences for the colon were found between VMAT and tomotherapy groups. Four dosimetric parameters were analyzed for the peritoneum, including the V_50.4_, V_40_, V_30,_ and V_25_ values. We also found that the 3DCRT group had higher peritoneum V_50.4_, V_40_, and V_30_ values than the VMAT and tomotherapy groups (*P* = 0.001, 0.002, and 0.013, respectively). In the analysis of peritoneum V_25_ values, the 3DCRT values were higher than those for the other two techniques, but this difference did not achieve significance (*P* = 0. 147). No significant differences in the dosimetric parameters for the peritoneum were observed between the VMAT and tomotherapy groups. Three dosimetric parameters were analyzed for the rectum, including the V_50.4_, V_40_, and V_30_ values. All three of these parameters were much higher for the 3DCRT group than for the VMAT and tomotherapy groups (*P* = 0. 003, < 0.001, and < 0.001, respectively). More importantly, the median rectum V_30_ values were 56.4% and 86.5% in the tomotherapy and VMAT groups, respectively. Tomotherapy further reduced the V_30_ value for the rectum compared with VMAT (*P* < 0.005). Figure 6 presents the isodose distributions in a representative T2N0 patient treated with VMAT and a T3N0 patient treated with tomotherapy, showing that the spiral delivery pattern associated with tomotherapy reduced the unnecessary dosing of the rectum. Three dosimetric parameters were analyzed for the bladder, including the V_50.4_, V_40_, and V_30_ values. The median V_50.4_, V_40_, and V_30_ values for the bladder in the 3DCRT group were 40.7%, 100%, and 100%, respectively. By contrast, the VMAT values were 2.0%, 28.0%, and 59.5%, respectively, and the tomotherapy values were 5.7%, 28.6%, and 50.5%. For all three parameters, the values for the 3DCRT group were much higher than for the VMAT and tomotherapy groups (*P* = 0. 001, < 0.001, and < 0.001, respectively). Similar to the finding for the colon and peritoneum, no significant differences were observed among the dosimetric parameters of the bladder between the VMAT and tomotherapy groups. Finally, five dosimetric parameters were analyzed for the bone marrow, including the V_50_, V_40_, V_30_, V_20_, and V_10_ values. Compared with VMAT and tomotherapy, we found that 3DCRT results in higher marrow V_50_, V_40_, V_30_, and V_20_ values (*P* < 0.001, < 0.001, < 0.001, and = 0.002, respectively) but not marrow V_10_ values. Similarly, no significant differences in dosimetric parameters for the bone marrow were observed between VMAT and tomotherapy.

**Table 8:** Dosimetric comparison of organs at risk (OARs) for the 3 treatment plans

	3DCRT (n = 9)	VMAT (n = 11)	Tomotherapy (n = 10)	*P* value
*Colon dose (%)*				
V_35_	34.7 [23.9–66.1]	13.0 [7.0–29.3]*	10.6 [3.5–17.1]*	0.002
V_25_	50.3 [41.2–74.3]	30.0 [18.5–53.0]^&^	30.4 [10.8–37.4]*	0.020
V_15_	64.9 [51.2–81.0]	68.0 [52.0–73.2]	57.1 [33.8–80.6]	0.796
*Peritoneum dose (%)*				
V_50.4_	35.8 [18.0–50.0]	3.6 [1.0–5.0]*	2.7 [0.5–5.2]*	0.001
V_40_	51.7 [28.0–73.0]	12.0 [7.0–21.0]*	*	0.002
V_30_	58.7 [35.2–79.7]	27.0 [20.0–37.0]*	23.6 [17.5–34.6]*	0.013
V_25_	66.7 [32.5–83.0]	45.0 [33.7–52.0]	36.6 [30.4–45.5]	0.147
*Rectum dose (%)*				
V_50.4_	24.4 [15.6,–52.6]	2.2 [0.0–13.4]*	5.3 [0.3–7.4]*	0.003
V_40_	97.5 [94.2–100.0]	37.0 [26.0–68.4]*	34.1 [26.2–40.6]*	< 0.001
V_30_	97.5 [96.3–100.0]	86.5 [69.3–90.0]	56.4 [49.3–66.0]*^§^	< 0.001
*Bladder dose (%)*				
V_50.4_	40.7 [31.2–46.7]	2.0 [1.0–14.0]*	5.7 [0.7–13.3]*	0.001
V_40_	100.0 [99.8–100.0]	28.0 [23.3–34.1]*	28.6 [21.3–46.1]*	< 0.001
V_30_	100.0 [100.0–100.0]	59.5 [52.0–71.0]*	50.5 [43.2–73.6]*	< 0.001
*Bone marrow dose (%)*				
V_50_	27.8 [24.1–29.2]	4.0 [2.7–5.0]*	5.5 [3.0–8.1]*	< 0.001
V_40_	44.0 [39.7–45.5]	16.0 [13.0–17.0]*	19.6 [16.9–23.0]*	< 0.001
V_30_	58.9 [54.8–62.9]	37.0 [34.2–38.5]*	39.2 [38.0–45.8]*	< 0.001
V_20_	88.8 [81.5–91.3]	70.0 [63.0–75.4]*	70.4 [67.1–73.3]*	0.002
V_10_	91.9 [84.4–95.6]	89.8 [87.0–94.0]	90.2 [87.1–92.7]	0.936

*3DCRT* three-dimensional conformal radiation therapy, *VMAT* volumetric modulated arc therapy, V_50.4_, V_40_, V_35_, V_30_, V_25_, V_15_ = volume receiving ≥ 50.4, ≥ 40, ≥ 35, ≥ 30, ≥ 25, ≥ 15 Gy, respectively

Differences were compared using the Kruskal–Wallis tests for continuous variables

Data are presented as the median [interquartile range]

*P < 0.05 versus 3DCRT in the Bonferroni post hoc test

^§^*P* < 0.05 versus VMAT in the Bonferroni post hoc test

^&^*P* = 0.09 versus 3DCRT in the Bonferroni post hoc

### RT-related toxicity and RT techniques

Table 9 presents the percentages of gastrointestinal (GI) and genitourinary complications associated with 3DCRT, VMAT, and tomotherapy. Acute Grade 2 or worse diarrhea for 3DCRT, VMAT, and tomotherapy occurred in 66.7% (6/9), 54.5% (6/11), and 10.0% (1/10) of patients, respectively. Tomotherapy substantially and significantly reduced the severity of acute diarrhea (*P* = 0.029). None of the patients suffered from Grade 4 diarrhea. Grade 2 or worse chronic colitis occurred in 22.2% (2/9) of the 3DCRT group, 18.2% (2/11) of the VMAT group, and 20.0% (2/10) of the tomotherapy group, with no significant differences noted between the three groups. For Grade 2 or worse acute cystitis, the incidences for the 3DCRT, VMAT, and tomotherapy groups were 33.3% (3/9), 63.6% (7/11), and 30.0% (3/10), with no significant difference noted between groups.

**Table 9:** The gastrointestinal and genitourinary toxicity of 3DCRT, VMAT, and tomotherapy

	3DCRT (n = 9)	VMAT (n = 11)	Tomotherapy (n = 10)	*P* value
*Diarrhea*				
Gr. 0/1	3 (33.3)	5 (45.5)	9 (90.0)	0.029
Gr. ≥ 2	6 (66.7)	6 (54.5)	1 (10.0)	
*Colitis*				
Gr. 0/1	7 (77.8)	9 (81.8)	8 (80.0)	0.975
Gr. ≥ 2	2 (22.2)	2 (18.2)	2 (20.0)	
*Cystitis*				
Gr. 0/1	6 (66.7)	4 (36.4)	7 (70.0)	0.230
Gr. ≥ 2	3 (33.3)	7 (63.6)	3 (30.0)	

*3DCRT* three-dimensional conformal radiation therapy, *VMAT* volumetric modulated arc therapy, *Gr*. grade

Data are presented as n (%)

## Discussion

Our study focused on the prognostic factors among non‐distant metastatic cervical cancer patients treated with definitive CCRT and compared RT-related toxicity among three different RT modalities.

Several studies examining the prognostic factors associated with cervical cancer have been published worldwide. The major identified prognostic factors include tumor size; pattern of invasion; tumor grade; pelvic nodal metastasis; age; race; socioeconomic status; severity of anemia; OTT; and the levels of biomarkers, such as hypoxia-inducible factor 1α (HIF-1α), vascular endothelial growth factor (VEGF), SCC Ag, and CEA. Huang et al. suggested that pretreatment SCC Ag > 40 ng/mL was an independent factor associated with para-aortic lymph node relapse, and pretreatment CEA levels have been identified as a risk factor for para-aortic lymph node recurrence, in addition to SCC Ag. Hong et al. also reviewed 401 patients with cervical cancer primarily treated with RT and concluded that pretreatment SCC Ag > 10 ng/mL was an independent predictor of poor disease-specific survival (DFS) [8–17]. Our study showed that patients with pretreatment SCC Ag > 10 ng/mL had worse PFS (HR 2.2, *P* = 0.041), LRRFS (HR 3.48, *P* = 0.038), and DMFS (HR 2.8, *P* = 0.045). In addition, the subgroup analysis in our study showed that pretreatment SCC Ag > 10 ng/mL was an effective predictor for DMFS in T1N0/T2N0 patients (HR 12.4, *P* = 0.066). These results suggest that even among patients with early-stage cervical cancer primarily treated with definitive CCRT, pretreatment SCC Ag might serve as a predictor for distant metastasis, which can aid clinicians in designing an effective treatment plan.

Radiotherapy combined with concurrent chemotherapy provides excellent curative effectiveness for patients with cervical cancer; however, RT-related toxicities are well known and can affect quality of life. RT-associated toxicity can occur at any time during treatment or even several months to years later. Acute complications can include diarrhea, desquamation, cystitis, nausea, and vaginitis, which may lead to the interruption of RT [18]. Late complications of radiotherapy may arise several months to years after pelvic irradiation, which can include radiation colitis, intestinal perforation, bowel obstruction, and vaginal stenosis, with profound effects on quality of life [19]. To reduce RT-associated side effects, fixed-field IMRT, VMAT, and tomotherapy have widely been used for pelvic irradiation, enhancing target dose conformity while reducing high-dose delivery to target-surrounding OARs [20]. However, few comparisons of dosimetric parameters and clinical outcomes have been reported among these various RT modalities. Lin et al. demonstrated a meta-analysis that combines six studies regarding a total of 1008 patients with cervical cancer to compare the efficacies and toxicities of IMRT with 3DCRT or conventional two-dimensional radiotherapy. And concluded that IMRT significantly reduced acute gastrointestinal and genitourinary toxicities as well as chronic genitourinary toxicity [29]. Guo et al. [21] reported that VMAT plans provided better protection of the rectum and bladder compared with fixed-field IMRT, but no significant differences were observed in the severity of complications. Our dosimetric data for the rectum in the VMAT group were similar to those reported by Guo et al. The rectum V_40_ and V_30_ values in their study were 47.39% and 82.12%, respectively, whereas, in our study, these values were 37.0% and 86.5%. However, the bladder V_30_ and V_40_ values in our study were lower than those reported by Guo et al., which may be due to differences in bladder preparation and target delineation. Hsieh et al. examined RT delivered by tomotherapy to the whole-pelvic area in 28 fractions totaling 50.4 Gy, followed by intracavitary brachytherapy, to treat locally advanced cervical cancer and reported decreased mean doses delivered to the rectum, bladder, and intestines compared with a conventional 4-field box plan [22]. Although the benefits of VMAT and tomotherapy for the treatment of patients with non-operative cervical cancer patients are generally accepted, little research has focused on dosimetric comparisons for OARs between these two plans. Our results suggested that even compared with VMAT, tomotherapy resulted in a significant reduction in the rectum V_30_ value, and was further reduced compared with 3DCRT. In addition to dosimetric parameters for the rectum, we also analyzed the bladder (V_50.4_, V_40_, V_30_), peritoneum (V_50.4_, V_40_, V_30_, V_25_), colon (V_35_, V_25_, V_15_), and bone marrow (V_50_, V_40_, V_30_, V_20_, V_10_); in addition to a reduction in the rectum V_30_ value, tomotherapy resulted in a reduced mean bladder V_30_ value, which may indicate a lower dose delivered to the bladder. Ultimately, these results indicated that the implementation of VMAT or tomotherapy reduced the delivery of high-dose radiation to normal tissues outside of the target volume, which was more apparent at higher radiation doses, which likely benefits adjacent OARs.

To date, few studies have examined the effects of small intestine volume in gynecological IMRT patients, and only one study has reported the rectal dosimetry associated with acute GI toxicity. Although the contribution of rectal dose parameters to acute radiation-induced GI toxicity remains a concern in patients treated for gynecological malignancies, most studies have primarily focused on the postoperative population. Therefore, the current study aimed to analyze the acute toxicities and rectal doses received by patients with cervical cancer treated with definitive CCRT. Roeske et al. analyzed 50 patients with gynecological malignancies who were treated with pelvic IMRT, approximately two-thirds of whom had received hysterectomies, and concluded that rectal dosimetry (range 35–49 Gy) was not a significant factor in acute GI toxicity. In that study, only half of patients (26/50) received concomitant chemotherapy, which might contribute to reduced radiosensitivity [23]. Deville et al. studied 67 patients undergoing postprostatectomy IMRT and noted that the minimum dose (D_min_) delivered to the rectum was marginally associated with acute Grade ≥ 2 GI toxicity (*P* = 0.05) [24]. Huang et al. examined the association between rectal dose and acute diarrhea in patients with gynecologic malignancies undergoing postoperative pelvic IMRT and showed that a mean rectal dose ≥ 32.75 Gy is an independent factor for the occurrence of Grade 2 or worse diarrhea [25]. The present study included patients with cervical cancer undergoing definitive CCRT using various RT treatment plans to study the dosimetric factors associated with acute radiation-induced GI toxicity and compared OAR dosimetry values between the 3 RT plans. Based on our study, the rectum V_30_ value is a meaningful predictor for the occurrence of acute diarrhea and chronic colitis, especially for acute Grade 2 or worse diarrhea.

The importance of the small bowel in acute GI toxicity is difficult to disregard, and a higher incidence of acute Grade 2 or worse diarrhea is generally considered to be caused by the increased irradiation of the small bowel among patients who receive whole-pelvic RT. However, studies examining the correlation between the volume of irradiation received by the small bowel and the incidence of acute RT-related diarrhea in gynecological IMRT patients are extremely rare. Roeske et al. reported a high-dose small bowel volume effect among pelvic IMRT patients (n = 50); Chi et al. found a high-dose (V_45_) small bowel volume effect among IMRT-treated patients (n = 32) with endometrial cancer. Huang et al. (n = 108) showed that the cumulative incidence of Grade 2–3 diarrhea among patients treated with 39.6 Gy delivered to small bowel volume < 60 mL and ≥ 60 mL were 33.3% and 63.4% (*P* = 0.001), respectively, and suggested that a small bowel volume of 39.6 Gy delivered to < 60 mL should be used as a constraint to alleviate acute RT-related diarrhea [23, 25, 26]. Our study used the peritoneum as a surrogate for the small bowel, which revealed that the peritoneum V_40_ has the potential to be used as a predictor for acute Grade 2 or worse diarrhea (OR 1.62, 95% CI 0.97–2.71, *P* = 0.068). A larger sample size remains necessary to verify the effectiveness of peritoneum V_4o_ as a predictor of diarrhea. We attempted to use the peritoneum instead of the small loop for dosimetric evaluations because the bowel wall is sometimes ill-defined and easily mobilized during non-enhanced CT simulations, making the contour process difficult and leading to low reproducibility.

The major toxicities associated with pelvic radiotherapy for gynecologic malignancies include complications involving the rectum, bladder, and bone marrow, leading to diarrhea, colitis, cystitis, and bone marrow suppression, which are categorized as acute or chronic toxicities depending on the time of onset. RT techniques continue to evolve, from 3DCRT to IMRT, and IMRT is considered to be an effective technique with a low incidence of acute toxicity [27]. However, whether VMAT or tomotherapy can further reduce the severity of RT-related toxicities is still debated. Few studies have compared clinical complications between fixed-field IMRT and VMAT or tomotherapy. Guo et al. compared the clinical toxicities and dosimetric parameters of VMAT and fixed-field IMRT in patients (n = 84) with cervical cancer who underwent radical CCRT and concluded that VMAT plans were superior to fixed-field IMRT plans in terms of the dosimetry values recorded for the V_30_ of the rectum and the V_40_ of the bladder, although no significant differences in acute and chronic complications were observed clinically [21]. Impressively, our study indicated that tomotherapy reduced not only the rectum V_30_ but also the severity of acute diarrhea compared with VMAT, indicating the potential to translate a dosimetric advantage into clinical benefits. Since we analyzed the acute diarrhea during EBRT rather than brachytherapy, the EQD2 of brachytherapy might not be an interference factor of acute diarrhea. In addition, there’s no significant difference in mean EQD2 of brachytherapy among three subgroups as shown in Table 1. The difference observed between the rectum V_30_ values between techniques in our study (tomotherapy vs. VMAT: 56.4% vs. 86.5%, *P* < 0.05) was larger than the difference reported in Guo’s study (VMAT vs. fix-field IMRT: 82.12% vs. 91.33%, *P* = 0.002), which may have contributed to the divergence in clinical outcomes. By contrast, no significant differences were observed among the 3 treatment plans for the occurrence of acute cystitis and chronic colitis. The bladder may not be as sensitive to radiation as the rectum, and a higher tolerance may reduce the occurrence of acute complications. Besides, chronic colitis might also be affected by brachytherapy and chemotherapy. Furthermore, the small sample size may not have been sufficiently powered to detect a difference between the groups.

Our study involved some limitations. First, this study was performed as a retrospective study. Unlike a prospective study, the present study inevitably includes a degree of selection bias, recall bias, and confounding effects, leading to a finite level of evidence. Second, the limited case number makes the results relatively tentative, and these findings must be confirmed in a larger sample. Third, only 30 of the 93 patients completed a dosimetric analysis because we initiated the dosimetric evaluation in the midterm of the study, although we did not artificially intervene in the case selection process.

## Conclusion

Pretreatment SCC Ag ≤ 10 ng/mL were associated with better PFS, LRRFS, and DMFS in patients with stage IB-IVA cervical cancer treated by radical CCRT and might serve as an effective predictor for DMFS in the T1N0/T2N0 subgroup. The V_30_ value of the rectum is an important dosimetric factor for acute diarrhea during pelvic EBRT. Compared with VMAT, tomotherapy reduced the V_30_ value for the rectum and consequently alleviated the severity of acute diarrhea.

## Data Availability

All data and materials have been presented in the manuscript.

## Abbreviations

CCRT: Concurrent chemoradiation therapy
EBRT: External beam radiation therapy
3DCRT: 3-Dimensional conformal radiation therapy
IMRT: Intensity-modulated radiotherapy
VMAT: Volumetric modulated arc therapy
RT: Radiation therapy
FIGO: International Federation of Gynecology and Obstetrics
ECOG: Eastern Cooperative Oncology Group
CT: Computed tomography
MRI: Magnetic resonance imaging
SCC: Squamous cell carcinoma
CEA: Carcinoembryonic antigen
CA125: Cancer antigen 125
AJCC: American Joint Committee on Cancer
IRB: Internal Review Board
AP: Anteroposterior
PA: Posteroanterior
CTV: Clinical target volume
PTV: Planning target volume
OAR: Organ at risks
CTCAE: Common Terminology Criteria of Adverse Events
OTT: Overall treatment time
LRRFS: Locoregional recurrence–free survival
PFS: Progression-free survival
DMFS: Distant metastasis–free survival
OS: Overall survival
PET: Positron emission tomography
EQD2: Equivalent dose in 2-Gy fractions
HR: Hazard ratio
CI: Confidence interval
OR: Odds ratio
HIF-1α: Hypoxia-inducible factor 1α
VEGF: Expression of vascular endothelial growth factor
SCC Ag: Squamous cell carcinoma antigen
DFS: Disease-specific survival

## References

[1] Arbyn M, Weiderpass E, Bruni L, de Sanjosé S, Saraiya M, Ferlay J (2020). Estimates of incidence and mortality of cervical cancer in 2018: a worldwide analysis. Lancet Glob Health.

[2] Akinyemiju T, Ogunsina K, Sakhuja S, Ogbhodo V, Braithwaite D (2016). Life-course socioeconomic status and breast and cervical cancer screening: analysis of the WHO’s Study on Global Ageing and Adult Health (SAGE). BMJ Open.

[3] De Felice F, Pranno N, Papi P, Brugnoletti O, Tombolini V, Polimeni A (2020). Xerostomia and clinical outcomes in definitive intensity modulated radiotherapy (IMRT) versus three-dimensional conformal radiotherapy (3D-CRT) for head and neck squamous cell carcinoma: a meta-analysis. In vivo (Athens, Greece).

[4] Marta GN, Weltman E, Ferrigno R (2018). Intensity-modulated radiation therapy (IMRT) versus 3-dimensional conformal radiation therapy (3D-CRT) for head and neck cancer: cost-effectiveness analysis. Rev Assoc Med Bras.

[5] Mell LK, Sirák I, Wei L, Tarnawski R, Mahantshetty U, Yashar CM (2017). Bone marrow-sparing intensity modulated radiation therapy with concurrent cisplatin for stage IB-IVA cervical cancer: an international multicenter phase II clinical trial (INTERTECC-2). Int J Radiat Oncol Biol Phys.

[6] Wang X, Shen Y, Zhao Y, Li Z, Gou H, Cao D (2015). Adjuvant intensity-modulated radiotherapy (IMRT) with concurrent paclitaxel and cisplatin in cervical cancer patients with high risk factors: a phase II trial. Eur J Surg Oncol J Eur Soc Surg Oncol Br Assoc Surg Oncol.

[7] Mell LK, Kochanski JD, Roeske JC, Haslam JJ, Mehta N, Yamada SD (2006). Dosimetric predictors of acute hematologic toxicity in cervical cancer patients treated with concurrent cisplatin and intensity-modulated pelvic radiotherapy. Int J Radiat Oncol Biol Phys.

[8] Rutledge FN, Mitchell MF, Munsell M, Bass S, McGuffee V, Atkinson EN (1992). Youth as a prognostic factor in carcinoma of the cervix: a matched analysis. Gynecol Oncol.

[9] Katz A, Eifel PJ, Moughan J, Owen JB, Mahon I, Hanks GE (2000). Socioeconomic characteristics of patients with squamous cell carcinoma of the uterine cervix treated with radiotherapy in the 1992 to 1994 patterns of care study. Int J Radiat Oncol Biol Phys.

[10] Shaverdian N, Gondi V, Sklenar KL, Dunn EF, Petereit DG, Straub MR (2013). Effects of treatment duration during concomitant chemoradiation therapy for cervical cancer. Int J Radiat Oncol Biol Phys.

[11] Hong JH, Tsai CS, Lai CH, Chang TC, Wang CC, Chou HH (2005). Risk stratification of patients with advanced squamous cell carcinoma of cervix treated by radiotherapy alone. Int J Radiat Oncol Biol Phys.

[12] Huang EY, Huang YJ, Chanchien CC, Lin H, Wang CJ, Sun LM (2012). Pretreatment carcinoembryonic antigen level is a risk factor for para-aortic lymph node recurrence in addition to squamous cell carcinoma antigen following definitive concurrent chemoradiotherapy for squamous cell carcinoma of the uterine cervix. Radiat Oncol (London, England).

[13] Huang EY, Wang CJ, Chen HC, Fang FM, Huang YJ, Wang CY (2008). Multivariate analysis of para-aortic lymph node recurrence after definitive radiotherapy for stage IB-IVA squamous cell carcinoma of uterine cervix. Int J Radiat Oncol Biol Phys.

[14] Jeong SY, Park H, Kim MS, Kang JH, Paik ES, Lee YY (2020). Pretreatment lymph node metastasis as a prognostic significance in cervical cancer: comparison between disease status. Cancer Res Treat.

[15] Kawanaka T, Kubo A, Ikushima H, Sano T, Takegawa Y, Nishitani H (2008). Prognostic significance of HIF-2alpha expression on tumor infiltrating macrophages in patients with uterine cervical cancer undergoing radiotherapy. J Med Investig.

[16] Loncaster JA, Cooper RA, Logue JP, Davidson SE, Hunter RD, West CM (2000). Vascular endothelial growth factor (VEGF) expression is a prognostic factor for radiotherapy outcome in advanced carcinoma of the cervix. Br J Cancer.

[17] Hong JH, Tsai CS, Chang JT, Wang CC, Lai CH, Lee SP (1998). The prognostic significance of pre- and post-treatment SCC levels in patients with squamous cell carcinoma of the cervix treated by radiotherapy. Int J Radiat Oncol Biol Phys.

[18] Lee J, Lin JB, Chang CL, Sun FJ, Wu MH, Jan YT (2018). Impact of para-aortic recurrence risk-guided intensity-modulated radiotherapy in locally advanced cervical cancer with positive pelvic lymph nodes. Gynecol Oncol.

[19] Dang YZ, Li P, Li JP, Bai F, Zhang Y, Mu YF (2018). The efficacy and late toxicities of computed tomography-based brachytherapy with intracavitary and interstitial technique in advanced cervical cancer. J Cancer.

[20] Hansen H, Høgdall C, Engelholm S (2014). Radiation therapy without cisplatin for elderly cervical cancer patients. Int J Radiat Oncol Biol Phys.

[21] Guo M, Huang E, Liu X, Tang Y (2018). Volumetric modulated arc therapy versus fixed-field intensity-modulated radiotherapy in radical irradiation for cervical cancer without lymphadenectasis: dosimetric and clinical results. Oncol Res Treat.

[22] Hsieh CH, Wei MC, Lee HY, Hsiao SM, Chen CA, Wang LY (2009). Whole pelvic helical tomotherapy for locally advanced cervical cancer: technical implementation of IMRT with helical tomotherapy. Radiat Oncol (London, England).

[23] Roeske JC, Bonta D, Mell LK, Lujan AE, Mundt AJ (2003). A dosimetric analysis of acute gastrointestinal toxicity in women receiving intensity-modulated whole-pelvic radiation therapy. Radiother Oncol J Eur Soc Ther Radiol Oncol.

[24] Deville C, Vapiwala N, Hwang WT, Lin H, Ad VB, Tochner Z (2012). Comparative toxicity and dosimetric profile of whole-pelvis versus prostate bed-only intensity-modulated radiation therapy after prostatectomy. Int J Radiat Oncol Biol Phys.

[25] Huang EY, Wang YM, Chang SC, Liu SY, Chou MC (2021). Rectal dose is the other dosimetric factor in addition to small bowel for prediction of acute diarrhea during postoperative whole-pelvic intensity-modulated radiotherapy in gynecologic patients. Cancers.

[26] Chi A, Nguyen NP, Xu J, Ji M, Tang J, Jin J (2012). Correlation of three different approaches of small bowel delineation and acute lower gastrointestinal toxicity in adjuvant pelvic intensity-modulated radiation therapy for endometrial cancer. Technol Cancer Res Treat.

[27] Salama JK, Mundt AJ, Roeske J, Mehta N (2006). Preliminary outcome and toxicity report of extended-field, intensity-modulated radiation therapy for gynecologic malignancies. Int J Radiat Oncol Biol Phys.

[28] National Institutes of Health (NIH). Common terminology criteria for adverse events (CTCAE) v4.03. https://ctep.cancer.gov/protocoldevelopment/electronic_applications/docs/CTCAE_4.03.xlsx. Accessed 14 June 2010.

[29] Lin YZ, Chen K, Lu ZY, Zhao L, Tao YL, Ouyang Y (2018). Intensity-modulated radiation therapy for definitive treatment of cervical cancer: a meta-analysis. Radiat Oncol.

